# Prospects for strain-specific immunotherapy in Alzheimer’s disease and tauopathies

**DOI:** 10.1038/s41541-018-0046-8

**Published:** 2018-02-27

**Authors:** Alice Bittar, Urmi Sengupta, Rakez Kayed

**Affiliations:** 1 Mitchell Center for Neurodegenerative Diseases, University of Texas Medical Branch, Galveston, TX 77555 USA; 2 Departments of Neurology, Neuroscience and Cell Biology, University of Texas Medical Branch, Galveston, TX 77555 USA; 3 Sealy Center for Vaccine Development, University of Texas Medical Branch, Galveston, TX 77555 USA

## Abstract

With increasing age, as the incidence of Alzheimer’s disease is increasing, finding a therapeutic intervention is becoming critically important to either prevent or slow down the progression of the disease. Passive immunotherapy has been demonstrated as a successful way of reducing large aggregates and improving cognition in animal models of both tauopathies and Alzheimer’s disease. However, with all the continuous attempts and significant success of immunotherapy in preclinical studies, finding a successful clinical therapy has been a great challenge, possibly indicating a lack of accuracy in targeting the toxic species. Both active and passive immunotherapy approaches in transgenic animals have been demonstrated to have pros and cons. Passive immunotherapy has been favored and many mechanisms have been shown to clear toxic amyloid and tau aggregates and improve memory. These mechanisms may differ depending on the antibodie's' target and administration route. In this regard, deciding on affinity vs. specificity of the antibodies plays a significant role in terms of avoiding the clearance of the physiological forms of the targeted proteins and reducing adverse side effects. In addition, knowing that a single protein can exist in different conformational states, termed as strains, with varying degrees of neurotoxicity and seeding properties, presents an additional level of complexity. Therefore, immunotherapy targeting specifically the toxic strains will aid in developing potential strategies for intervention. Moreover, an approach of combinatorial immunotherapies against different amyloidogenic proteins, at distinct levels of the disease progression, might offer an effective therapy in many neurodegenerative diseases.

## Introduction

The past two decades witnessed significant improvements in the fields of several major diseases such as cancer and AIDS at the level of clinical survival rates. However, this has not been the case for Alzheimer’s disease (AD). Clinical trials continue to fail and death rates continue to increase.^1^ According to the Alzheimer’s association, the number of people with AD in 2017 have exceeded 5.5 million only in the United States, and the population is expected to triple by 2050.^2^ Most of this population live with Alzheimer’s dementia at the age 65 or older and are expected to die before the age of 80.^3^

Therapeutic intervention of age-related neurodegenerative diseases has been a major hub of research for several years now. Increasing age owing to the multiple complex mechanisms being involved in these diseases has increased the difficulty of finding a therapeutic intervention. Both scientific and pharmaceutical fields together have been trying to find therapy with higher sensitivity and efficacy. The majority of earlier work was confined to link Aβ plaques to the disease severity, which became later disapproved as the plaque burden did not correlate with the disease state. Under the light of amyloid cascade hypothesis, the immunotherapeutic research was mainly focused on only Aβ-amyloid for many years. However, continuous attempts of successful amyloid immunotherapy and their consecutive failure rate have altered the attention to another major protein, tau.^4^ Unlike Aβ plaques, tau constituting neurofibrillary tangles (NFTs), another pathological hallmark of AD, was well indicative of the degree of cognitive decline. Studies showing the differential distribution of this protein in the pre- and post-synaptic compartments in the disease state suggested its translocation from its physiological localization in the axon to a more pathological localization in the dendritic spines.^5^

With all the accumulated knowledge over the past three decades, the mechanisms underlying the pathophysiology of the disease are still a mystery and scientists are not able to settle on a common hypothesis regarding the main cause of the disease.^6,7^ This may be because of the highly complex nature of the disease. Nevertheless, the question that forces itself after decades of research and clinical trials is that: are we aiming at the right target(s) for potential therapeutics?

This review will cover the most recent findings concerning the progression of amyloid and tau pathology, targeting Aβ and tau by immunotherapy, and the option of combination therapy as potential treatment for AD.

## Amyloid cascade hypothesis progression and tau

Initially, the amyloid cascade hypothesis was the prevailing view over the field of AD. Most researchers thought that Aβ plaques accumulation following amyloid precursor protein (APP) misprocessing is the main cause behind neurodegeneration. It was also thought that the severity of the disease could be reduced by decreasing extracellular Aβ plaques load. Many immunotherapy approaches against Aβ plaques strongly supported this notion in cell culture models^8–10^ and in vivo animal models studies.^11–13^ However, results from a plethora of studies in animal models and clinical trials challenged the “golden hypothesis”. It was shown that Aβ plaques load in human brains does not correlate with the level of cognitive impairment,^14^ and that dementia precedes plaque deposition by a long time in animal models.^15,16^ This strongly indicated that Aβ plaques may be the result and not the cause of the neurotoxicity, and that the smaller soluble protein aggregates and their interactions were the drivers of pathology propagation. Furthermore, many human brains were shown to possess a high Aβ plaque load with no dementia, thus refuting the causative relationship between Aβ accumulation and neurodegeneration.^17^ However, keeping all this in mind, Aβ protein is part of the pathology and its role in AD development and treatment cannot be ignored.

**Fig. 1:**
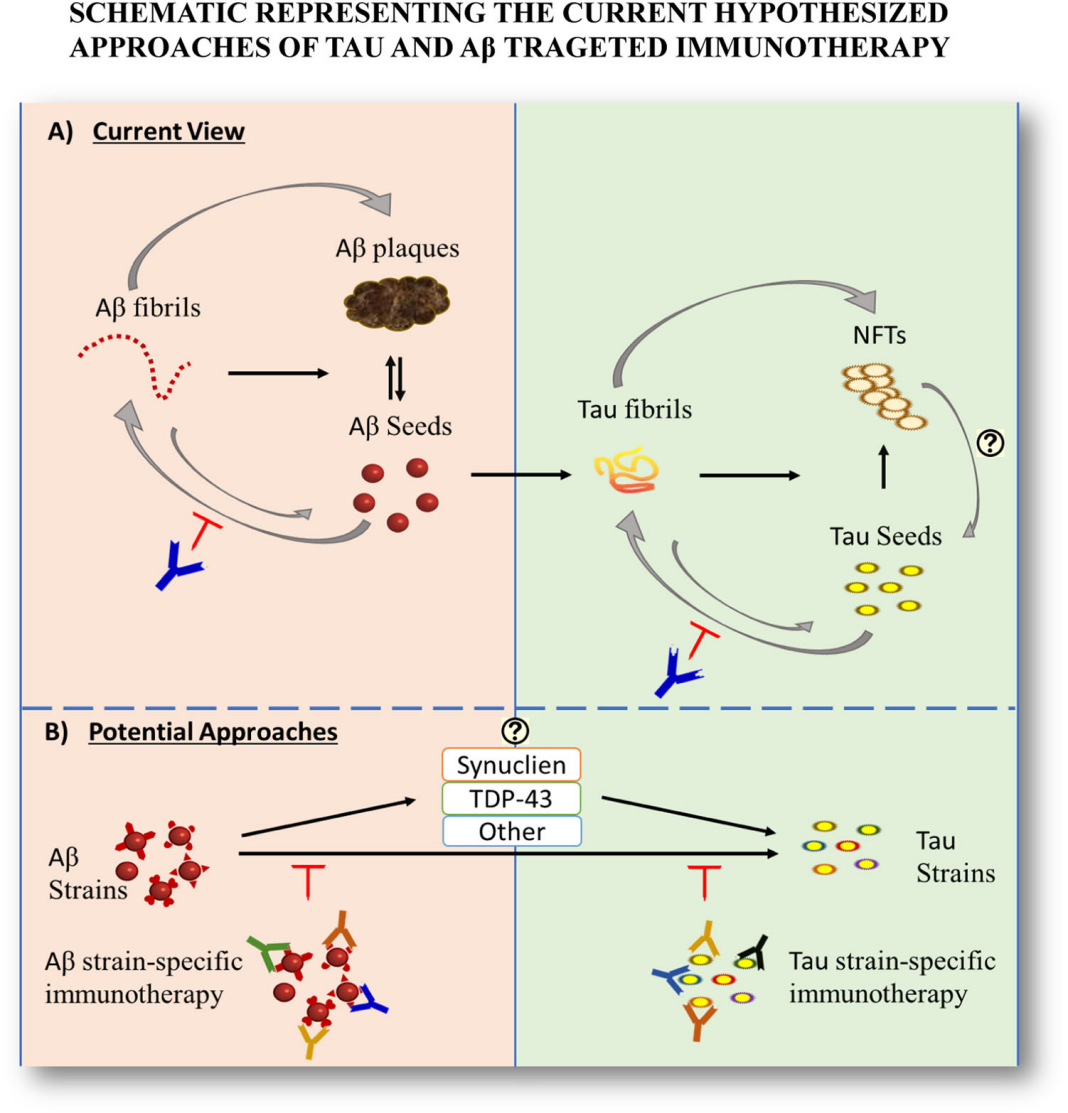
Immunotherapeutic approaches targeting amyloidogenic seeds and secondary amyloidosis thus, preventing disease progression. **a** A representation of the current view of the relationship between tau and Aβ toxic species, which are being targeted by passive immunotherapy. **b** Recent research suggests that effective immunotherapies may require targeting multiple strains/conformers formed by different amyloidogenic proteins

Another pathological hallmark of the disease is tau protein. Tau was first recognized in the form of NFTs in 1998 by Braak and others.^18^ It was not until recently though that the field have launched more extensive investigations of tau protein species and its toxic forms.^19–21^ So far, it has been shown, that the extent and spatial progression of cognitive dysfunction and neuronal death correlate well with tau pathology load and spatial distribution in the brain, unlike Aβ load.^22–24^ More importantly, it was shown that tau pathology temporally overlaps with the start of dementia.^18^ This suggests a direct causative relationship between tau pathogenesis and neuronal dysfunction, a relationship that failed to be established between Aβ load and dementia.^25^ In addition, the most recent studies indicate that tau oligomers, and not the larger aggregates, are the toxic species and the cause of neuronal death.^26–30^

Bearing in mind the above findings, recent studies have revisited the amyloid cascade hypothesis by adding tau into the picture and suggesting that tau and Aβ converge into a cascade that eventually leads to neurodegeneration.^31^ More evidence have emerged supporting the concept of tau and Aβ interaction, especially after it was shown that the reduction of tau led to a reduction in Aβ load in P301L model.^32^ In addition, an interesting study showed that targeting soluble Aβ alone was not sufficient, and that targeting soluble tau in addition to Aβ was necessary to reduce neurodegeneration.^33^ The facts that Aβ immunotherapy trials showed little promise and low safety profiles, and that tau pathology correlated better with neurodegeneration and dementia have made tau a more attractive target for therapeutic approaches. Still we must be mindful of physiological tau and design therapies that specifically target toxic tau species and avoid side effects.

## Tau in animal models

Most amyloid AD animal models rely on Mendelian genetic mutations in the APP pathway or *PSEN* genes, where in reality, familial AD cases represent <1% of the AD population worldwide.^34^ Therefore, sporadic AD has not been well addressed by the available amyloid transgenic models.^35^ On one hand, most Aβ-based animal models, even the multiple *APP* and *PSEN* genes mutation models like the 5 × FAD, show only Aβ and no NFT pathology, unlike human post-mortem tissue.^36–38^ On the other hand, the convincing evidence that tau is imperative for neurodegeneration has led to the development of several tauopathy mouse models like JNPL3, pR5, and PS19 expressing human tau mutation P301L and P301S^39–41^ that lack Aβ pathology. These models confirmed that tau loss of function or gain of function alone can lead to neurodegeneration without the presence of Aβ pathology.^42^ As a result, some combinatorial models have been developed and succeeded in recapitulating human pathology. For example, the 3xtg-AD mouse model was generated by expressing human tau with P301L mutation combined with an APP transgene with Swedish mutation expressed in a PSEN1 knock-in mouse.^34,43^ In another model, a cross-breed between the APP transgenic Tg2576 and JNPL3 mouse models, the injection of a long form of Aβ_42,_ induces NFT formation and tau phosphorylation in areas projecting to the injection site.^34,44^ Findings from such combinatorial models have made clear the interaction between tau and Aβ, although it is not entirely clear which protein (tau or Aβ) is downstream of the other. One hypothesis suggests that NFT formation occurs downstream of Aβ processing or caused by Aβ aggregation.^45,46^ Specifically, some studies suggest that the misfolding and aggregation of Aβ protein triggers the hyperphosphorylation of tau protein and its subsequent aggregation into NFTs.^47^ If tau aggregation is downstream of Aβ, why target tau? why not target Aβ being the source of the disease and stop any downstream effects? In addition to the need of targeting Aβ seeds very early, a counter hypothesis recently emerged suggesting that tau is actually upstream of Aβ. Tau, being a microtubule-binding protein that orchestrates protein transport along the neuron, when malfunctioning, it self-propagates and leads to the aggregation of Aβ and possibly other proteins.^48^

That being said, it seems that the interaction between tau and Aβ is bidirectional with feedback loops; and targeting either Aβ or tau alone is probably not the right approach towards an optimum therapeutic.^49^ However, all what we are looking for is a trace of hope, a slight drug or therapeutic efficiency in clinical trials on which more efficient approaches can be built. Once obtained, further plans, like combinatorial therapies, might be ventured upon to reach optimum efficiency. Further elaboration on combinatorial therapy approaches will be presented later in this review.

Away from Mendelian genetic mutations in amyloid or tau, apolipoprotein E (*APOE*) gene was identified to be one of the most compelling risk factors for sporadic AD based on genome-wide association study (GWAS) studies.^50^ *APOE* gene mutation animal models express not only Aβ pathology, but also tau NFTs similar to human AD, and might be the closest to sporadic AD cases.^51^ APOE4 gene knockouts showed significantly reduced Aβ burden.^52^ Also, it was shown that APOE mutations contribute to hyperphosphorylation and accumulation of tau as early as 1 month of age in a targeted replacement APOE mouse model. Aβ pathology appears much later in the same APOE transgenic model.^53^ APOE mouse model findings support the notion that targeting tau early on in high-risk patients; patients with APOE mutations, might stop the downstream tau pathology and help reduce Aβ pathology in turn. All immunotherapy clinical performed on *APOE4* gene carriers so far target Aβ and show no improvement with antibody treatment (clinical trials ID; NCT00575055 and NCT00574132, clinicaltrials.gov). However, no trials are currently in development to target tau in APOE carriers yet. Since APOE carriers are at higher risk for developing AD, they might be a better population to investigate the effect of tau immunotherapy on cognitive decline.

Triggering receptor expressed on myeloid cells 2 (TREM2), a protein that is expressed on microglia and aids in the humoral immune response, was linked to neurodegeneration through GWAS studies. TREM2 receptor, in its normal state, forms a protein complex that is required for microglial activation and is imperative for clearing macromolecules like cellular debris and protein aggregates.^54^ Mutation of this receptor causes impaired clearance of pathogenic protein aggregates such as Aβ plaques thus increasing the risk for AD. Not only through Aβ pathogenesis, tau and p-tau CSF levels were also associated with *TREM2* gene.^55^ Therefore, it is expected to be found that the increase in TREM2 cerebrospinal fluid (CSF)-soluble fragments level temporally overlaps with the onset of clinical AD dementia.^56^ In addition, TREM2 mutations have been shown to directly affect the effectiveness of immunization, especially that of an active approach; TREM2 deficiency have been shown to decrease the uptake of Aβ-bound antibodies by microglia, decreasing in turn Aβ clearance.^57^ Among the several *TREM2* gene variations, R47H, D87N, and R62H were specifically linked to increased risk of AD and have been shown to act as early biomarkers for the disease.^58–63^ Most of the TREM2 risk variants have been found to be associated with microglial activation, which supports the importance of the innate immune response, and thus immunotherapy in AD and neurodegeneration.^63^ In addition, knowing that TREM2 research is still in its infancy, and that TREM2 mutation carriers are relatively rare within the AD population,^64^ considering TREM2 genetic variations/manipulation in Aβ or tau immunotherapy translational studies and clinical trials might provide a window into developing novel ways to advance immunotherapy approaches and increase its efficiency.^64^

Immunotherapy is one of the most common and thoroughly investigated potential therapeutic strategies in the field of Alzheimer’s and other neurodegenerative diseases.^65^ This is because of the specificity and selectivity it potentially offers in eliminating only the toxic species leaving the cell’s normal proteome intact. Most basic research, preclinical and clinical studies in the past two decades have been focusing on enhancing immunotherapy, whether passive or active, to better target either Aβ or tau protein aggregates.^66^ Immunotherapy in animal models have been shown to prevent Aβ or tau protein aggregation and deposition in the brain, promote protein aggregates clearance, and consequently decrease the levels of neurodegeneration and recover cognitive function.^66^ However, with clinical trials using Aβ immunotherapy failing to attain the set endpoints, current clinical trials using tau immunotherapy are underway.

## Mechanism of antibody action in passive immunotherapy

Both active and passive vaccination strategies attempt to target different phosphorylation sites, aggregation, and spreading states of tau in the brain. This process involves various mechanisms by which the deleterious tau protein effects are removed. Although, a number of studies have shown positive implications of tau immunotherapy in various transgenic animal models, the mechanism of action is not yet fully elucidated and thus is the center of an immense area of active research.^67–70^ Multiple approaches have been undertaken to prevent or delay the disease progression.^71, 72^ We have shown passive immunization in JNPL3 animals (a tauopathy animal model) with TOMA antibody specific for tau oligomers, where oligomers can be cleared up via a peripheral pathway, as also shown by other studies.^73, 74^ Sigurdsson laboratory published a series of findings indicating that active and passive immunization of tau can reduce levels of aggregated tau. Upon active immunization with the pathogenic tau379–408 and hyper-phosphorylated tau at Ser396 and 404 sites, levels of tau aggregates in JNPL3 animal model and htau/PS1(a tangle model) decreased with a reduction in cognitive decline in the latter model.^69, 75^ It has also been suggested that autophagy plays a role in clearing up tau aggregates upon internalization of tau-antibody complexes into the cells.^76^ Studies on primary neuronal cell cultures from JNPL3 animal model show that an antibody-based therapy for pathogenic tau clearance depends on the internalization or uptake of the antibody by the cells. In this study, the researchers had demonstrated that the uptake of antibody 4E6G7 is facilitated by a clathrin-mediated endocytic pathway via FcγII/III receptors by pharmacologic inhibition of both clathrin and FcγII/III receptors.^77^ Consistent with this observation, another recent study has shown that TRIM21 (tripartite motif protein 21), a receptor for Fc domain of antibody expressed in the cytoplasm plays a role in directing antibody-mediated tau clearance. In this study, exogenous tau seeds and antibody assemblies were added to the cells. Upon internalization of these complexes into the cells, intracellular TRIM21 led to a neutralizing mechanism of the tau seeds via strongly binding to the antibody, thus blocking the aggregation of native tau protein by the seeds.^78^ Passive immunization with tau antibodies directed against different phosphorylation sites showed reduced burden of hyper-phosphorylated tau aggregates and cognitive improvements.^70,79, 80^ It is suggested that aggregated tau is taken up by the phagosome, which also indirectly activates the uptake of antibodies via endocytosis. Fusion of these phagosomes with endosomes carrying antibodies is followed by their fusion with lysosome, subsequently leading to the degradation of tau aggregates. As an alternative, antibodies diffuse through the neuronal membranes and later bind to the tau aggregates. The entire complex is then subjected to degradation via endosome–lysosomal pathway.^81^ A study suggested that the uptake of antibody occurs via binding with its antigen present on neuronal membrane. In a passive immunotherapy with MAb86, an antibody targeting tau-pSer422, binds to tau-pSerS422 that is located in the lipid rafts of neuronal membrane. The Antibody bound to tau is then endocytosed into the cell leading to the degradation of bound tau via lysosomal pathway, and thus protecting cell’s internal clearance mechanism.^82^ One of the proposed mechanisms of Aβ immunotherapy is peripheral sink mechanism that relies on the clearance of Aβ sequestered by peripheral antibodies.^83^ The same has been suggested for tau immunotherapy where tau is cleared up from the brain into the peripheral system.^73,74, 84^ According to the prion-like hypothesis, when toxic form of tau is released into the extracellular space, it is believed to propagate from cell to cell. Therefore, apart from targeting intracellular tau aggregates, many tau immunotherapy research studies have focused on clearing up extracellular tau aggregates to prevent their spreading. A number of detailed structural studies are being performed to determine the internalization mechanisms of antibodies.^85, 86^ This also draws the attention to the various conformations that the aggregated tau can take off where the epitopes for the antibodies can be either exposed or buried in the aggregate. In this respect, development of strain-specific antibodies are of great significance.

## Active or passive?

We have learned that active and passive immunotherapy approaches each have advantages and disadvantages. Active immunotherapy, which exploits the body’s own immune system, is more natural and long-lasting. However, it results in a high risk of an autoimmune reaction and is limited in terms of epitope choice and specificity.^87, 88^ On the other hand, while passive immunotherapy is short-lasting and thus requires continuous antibody injections,^89^ antibodies can be designed with a high level of specificity to target subspecies or strains of the desired protein.^48^

It has become more convincing that tau oligomers, and not NFTs, are the most toxic species and that they directly correlate with dementia. After all, it has been shown that neurons can survive with a load of NFTs^19^ and that tau oligomers better correlate with the Aβ-mediated neuronal toxicity.^26,90, 91^ NFTs are regarded as a ‘‘result’’ rather than a ‘‘facilitator’’ of neuronal death. To better target tau oligomers, passive immunotherapy is needed. That is especially after finding that tau oligomers may exist in several isotypes or strains that might contribute to different tauopathies and cognitive dysfunctions.^92^ That being said, specificity become a priority in developing an immunotherapeutic. In our laboratory, we have developed several monoclonal antibodies specific for tau oligomers. We were able to show that these antibodies protect the mice from cognitive decline at the age of 5 to 8 months.^93, 94^ These results are promising and hold great potential for clinical trials. It is true that the amount of administered antibody reaching to the central nervous system (CNS) is insufficient due to biological barriers such as blood–brain-barrier, blood–cerebrospinal fluid-barrier, blood–retinal-barrier, and blood–spinal cord-barrier. These barriers maintain CNS homeostasis by limiting the circulation of molecules from peripheral circulation.^95^ Among these barriers, blood–brain-barrier (BBB) is the most stringent one allowing only 0.1–0.2 % of circulating antibodies to the brain.^96, 97^ While there is a controversy over whether or not the BBB is compromised in AD patients, novel techniques have risen to enhance antibody delivery into the CNS such as taking advantage of transferrin receptor (TfR)^98–100^ intranasal delivery,^101^ and others.

## Specificity vs. affinity

Only a handful of studies have been performed to investigate the importance of specificity in tau immunotherapy. A study by Petry et al.^102^ characterized an array of commercial and non-commercial monoclonal and polyclonal antibodies in terms of specificity to their corresponding tau isoform. To their surprise, a degree of inspecificity was associated to each of the tested antibodies. Even MC1 monoclonal antibody, despite showing the highest specificity among the tested antibodies, still showed a non-specific band on the western blot. However, this was referred to the denaturation of the conformational tau recognized by MC1 in the sodium dodecyl sulfate polyacrylamide gel electrophoresis. It should be noted that this study depended on western blot analyses, leaving room for non-specific observations. Another study comparing a similar array of antibodies in terms of affinity and specificity by utilizing immunohistochemistry showed that the antibodies with the highest affinity showed the lowest tau clearance, whereas those with the highest specificity were the most effective in reducing pathological tau levels. This highlights the importance of specificity over affinity in terms of tau clearance.^103^

Another study investigating two antibodies specific to the same tau epitope claims that antibody affinity alone is sufficient to predict its efficacy in tau clearance and protection from neurotoxicity. Similar to previous studies, they found that high-affinity antibodies are not effective in clearing soluble tau species and protecting neurons from toxicity, whereas low-affinity antibodies are.^104^ This was explained by the ability of high-affinity antibodies to cling mostly to non-soluble tau aggregates, i.e., the non-toxic species, leaving the soluble ones intact and thus not protecting the neurons from toxicity. When choosing between a low-affinity antibody and a high-specificity antibody, it becomes difficult as other factors also come into play such as method of immunotherapy, the degree of antibody internalization and whether the targeted tau species resides intracellular or extracellular.

## Potential benefits and side effects of targeting different tau species

Numerous studies of immunotherapy targeting phosphorylated tau yielded varying degrees of results. First of all, active immunotherapy with phospho-tau epitopes led to deleterious effects.^105, 106^ On the other hand, passive immunization studies using different antibodies that target different phospho epitopes on tau protein were performed in different transgenic animal models, at different stages of the disease pathology and moreover, via different routes for antibody administration. Among them, some studies showed behavioral improvements in animal models with both a presence and absence of the disease pathology. Whereas, another study demonstrated no difference in the survival of transgenic vs. control animals upon passive immunization, even though, there was reduction in observed tau pathology.^107^ More research is required to determine if the phospho-tau epitopes are necessary for tau seeds formation. Studies from our and multiple other laboratories have emphasized the role of oligomeric forms of tau protein as the toxic intermediates, which can be effectively targeted via passive immunotherapy using strain-specific antibodies.^73^ A potential side effect of tau immunotherapy could result from targeting normal physiological tau by the delivered antibody. Therefore, to rule out the side effects, the amount of total tau in vaccinated animals should not be altered. Nevetheless, astrogliosis has not been not observed, thus further confirming the safety of the studies.^69^ In addition, antibody-mediated immunotherapy targeted for extracellular large aggregates like amyloid plaques or Lewy bodies can cause breakdown of these aggregates into smaller entities. These smaller entities may cause edema or autoimmune response, which are also considered as a disadvantage of immunotherapy.^89^ Albeit the success of immunotherapy in transgenic animal models, this success could not successfully be reproduced in clinical trials. As the same is applicable for tau immunotherapy, the consideration that poses a major challenge to immunotherapy is accurately targeting the neurotoxic species of tau, which hinders the process of an effective drug development.^108^ Therefore, achieving modulators/antibodies that are specific to the neurotoxic species is of foremost importance.

## Effects of tau immunotherapy on amyloid pathology

Tau pathology has been reported to be a downstream mediator of Aβ pathology by several studies.^40, 107^ Previously, our laboratory has demonstrated that passive immunization of an amyloid animal model, Tg2576, with TOMA antibody (a tau oligomer-specific monoclonal antibody) reduces the level of toxic tau oligomers, which eventually decreases the burden of Aβ*56 aggregate.^109^ Thus, this data is consistent with the hypothesis that tau toxicity increases Aβ-pathology via a feedback loop.^31^ It is also hypothesized that the pathogenic conformers of both tau and Aβ interact and induce varying degrees of downstream effects in different patients.^107^ Similar to our study, another recent study of passive immunization with N-terminal tau antibodies demonstrated a decrease in hyper-phosphorylated and total tau levels in a dose-dependent manner, rescuing short-term episodic impairments of memory. Moreover, it also reduced APP, Aβ level and amyloid plaque burden.^110^ These studies demonstrating positive effects of tau immunotherapy reducing amyloid burden provide a promising avenue to target both tau and Aβ immunotherapies in combination.

## Targeting tau strains by immunotherapy

Recent advances in the research of neurodegenerative disorders have now established that a single protein may also occur in multiple neurotoxic forms in a particular proteinopathy. Aβ protein forms strains with different conformations in vitro^111^ and in vivo*.*^112^ Another study demonstrated the formation of a disease-specific strain of aggregated α-synuclein different from the one occurring in Parkinson’s disease.^113^ These studies suggest the importance and necessity to better understand the occurrence and the difference between strains of these amyloidogenic proteins in various clinical subtypes of AD pathology.

Human embryonic kidney (HEK293) cells were stably transfected to express different strains of tau which were shown to propagate their characteristics through generations. Upon inoculation of these tau strains into animal brains, the latter exhibited different tau pathologies that are disease-specific. This implies that variation in tau protein aggregates is associated with different brain regions in different neurodegenerative disorders.^114, 115^ Mostly, tau pathology has been extensively studied as an “inter-disease phenomenon” shown in tauopathies including AD, frontotemporal dementia with Parkinsonism linked to chromosome-17, progressive supranuclear palsy, Pick’s disease, and cortico-basal degeneration.^116–119^ With the advancement in tau imaging ligands, it has been feasible to visualize different entities of tau aggregates in living patients with tau pathology. A recent study combined two such widely used chemical agents, ^11^C-PBB3 and ^18^F-AV-1451, which allowed the visualization of a diverse number of tau fibril strains by positron emission tomography (PET) imaging.^120^ This envisages the necessity to look at tau as an “intra-disease” pathology as well. Besides tau pathology occurring in different tauopathies, same tauopathy may involve different strains or conformers of tau. A better understanding of these diverse tau conformers will shed light on how to develop more stream-lined and disease-specific therapy.

## Aβ immunotherapy and clinical trails

So far, it cannot be ruled out that targeting Aβ will be helpful in ameliorating neurodegeneration. However, several clinical trials utilizing immunotherapy against Aβ argue the opposite. One of the first clinical trials targeting Aβ was performed using AN1792, synthetic amyloid-β peptide (Aβ_42_) that induces the generation of antibodies against Aß by active immunotherapy. AN1792 was tested in a Phase 2a trial, however, the trail was halted when around 6% of treated patients developed meningoencephalitis.^121^ Even when examining the post-mortem brains from patients who did not develop meningoencephalitis from the same study, no protective cognitive effect was observed over the duration of the study (12 months) despite significant reduction in Aβ plaque load locally in the brain. Interestingly, a follow-up study, done 3.6 to 4.6 years after immunization with AN1792, found significant improvements at the level of brain volume and cognitive decline in immunized vs. placebo group. These improvements were observed in 68% of antibody responders. However, antibody responders were only around 19.6% of patients immunized with AN1792, which is a low turnout.^122^ Intriguingly, AN1792 was shown to target and reduce tau aggregates in neuropil threads and dystrophic neurites, however, tau aggregate formation in neuronal cell bodies was not affected. This suggests that targeting extracellular large tau aggregates is not beneficial either. Considering that the patients enrolled in this study were moderately-to-severely demented, the results of the AN1792 clinical trial suggest that targeting/reducing Aβ plaque load in the later stages of the disease does not produce consistent cognitive improvements optimum for a therapeutic approach.

Phase 1 trials of another active immunotherapeutic agent, CAD106, found it to be safe and well-tolerated, with no signs of meningoencephalitis in a 58-subject cohort.^123^ Two Phase 2a studies with open-label extensions also indicated a favorable safety profile.^124^ Therefore, CAD106 remains in development.

Several passive immunotherapeutic agents have been developed, including bapineuzumab and solanezumab. Bapineuzumab is a passive immunotherapeutic agent that targets the *N*-terminus of Aβ. It failed in clinical trials due to its poor safety profile. The agent was tried in mild-to moderate AD patients, some of which were *APOE4* gene carriers. The treatment was shown to increase the occurrence of vasogenic edema and expressed poor clinical efficacy.^125–127^ The drug was abandoned in the middle of Phase 3 clinical trial. Solanezumab, on the other hand, a humanized IgG1monoclonal antibody that targets soluble monomeric Aβ already passed Phase 1 and 2 trials and had a favorable safety profile. However, Phase 3 trials did not show any clinical efficacy either and thus solanezumab was also abandoned until last year (ID: NCT00905372 and NCT00904683).^128^ Solanezumab is back in clinical trials (ID NCT02008357, clinicaltrials.gov), however, only in early onset AD patients with a genetic mutation. The study includes patients from the age range 18 to 80 years old, thus comprising of those who are no yet demented to those with severe dementia. This clinical trial aims at better understanding the sequential order of pathological events in relation to CSF-soluble Aβ-42. So far, what the study has found is that Aβ-42 decreases in the CSF 25 years, and Aβ deposition starts 15 years before cognitive decline. This is an interesting finding because it narrows down drug targeting to Aβ-42. In addition, solanezumab was given to patients with mild-to-moderate AD. The patients tolerated a dose up to 400 mg weekly for 12 weeks. Levels of soluble unbound Aβ-42 increased in CSF in a dose-dependent manner unlike results obtained after AAB-003 (NCT01369225) and bapineuzumab in recent clinical trials. Nevertheless, cognitive function assessments showed no change after 12 weeks of treatment. The trial is still ongoing, looking for a shred of hopeful outcome (ID NCT01760005).^129^ Aducanumab, another monoclonal antibody currently in phase III of clinical trials, (NCT02477800 and NCT02484547) produced some promising results in phase I study (NCT01677572), which was performed on a group of mild and prodromal AD patients with and without APOE4 mutations. Data from phase I were very promising for the first time after several failed clinical studies (mentioned above). Aducanumab was shown to decrease soluble and fibrillar forms of Aβ in several brain regions associated with cognitive decline (such as frontal and parietal cortices, posterior cingulate cortex, lateral temporal, and sensorimotor cortex, as well as anterior cortex), and slow down cognitive decline in clinical dementia rating-sum of boxes and mini mental state examinations 54 weeks after treatment in a dose-dependent manner.^130^ The doses used ranged from 1 to 10 mg/kg. No increase in amyloid-related imaging abnormalities (ARIAs) from baseline ARIAs were reported, except in APOE carriers. Up to 55% of APOE carriers who received a dose of 10 mg/kg showed increase in ARIAs.^131^ Other side effects reported include dizziness, headaches, and diarrhea. Because of the reported promising results, phase III efficacy studies are currently ongoing and will include a large pool of 1350 patients with mild cognitive impairment or mild AD to determine the optimum dose of Aducanumab. The study will end in 2022 (NCT02477800).

Despite the fact that these studies were performed only on mild AD patients, results from phase I of aducanumab are a breakthrough. This is because the studies showed for the first time that the reduction of amyloid burden slows down cognitive decline. However, no further investigation was done on the soluble tau levels in these individuals. As mentioned earlier, Aβ active immunotherapy trials have suggested an interaction between Aβ and tau, and therefore, the decrease in cognitive decline after Aducanumab cannot be attributed to the reduction of Aβ alone. Nevertheless, this study might be very helpful for future novel drug design and treatment plans in patients with moderate to severe AD.

Gantenerumab, the first passive fully anti-human anti-Aβ antibody, interacts with Aβ fibrils, aggregated, and is intended to recruit microglia, activate phagocytosis and degrade Aβ-plaques as well as prevent the formation of new Aβ plaques.^132^ Phases I through II of several studies done on gantenerumab in several countries showed great efficacy in terms of decreasing Aβ load, however, ARIAs and focal inflammation were observed in the higher doses used. No clinical data is available on the effect of gantenerumab on the cognitive decline yet. Clinical trials continue until the end of 2018 (NCT02051608).^132^ Like aducanumab, gantenerumab studies recruit patients with mild cognitive impairment, prodromal AD, and mild AD. It had been noted, however, that if promising signs of cognitive improvement are observed, gantenerumab will be utilized in studies on patients with moderate AD, as well as in combinatorial treatment with Roche BACE inhibitor RG7129. The mentioned combinatorial studies have been performed on an aggressive Aβ mouse model, where the combination between gantenerumab and Roche BACE inhibitor RG7129 showed a positive additive effect.^133^ Further information and studies are needed to determine the efficacy of gantenerumab either alone or in a combination therapy approach.

## Tau immunotherapy and clinical trails

Not much has been determined concerning tau immunotherapy clinical trials so far, and the first results will help better shape the future path. However, the past two decades of Aβ immunotherapy studies were helpful in setting protocols and research strategies that will better guide the use of tau immunotherapy.^134^ Many questions still need to be addressed before developing the optimum tau immunotherapeutic. Much to be done in this new era of AD research. Although tau’s role in AD is now well established, very few tau immunotherapy studies have made it to clinical trials, as many are still in the safety phase I stage.

No passive antibodies have been made it to interventional clinical trials yet, however, a few are in the process. AADvac-1, an active vaccine with a natural truncated form of tau recently moved to phase II clinical trials (ID: NCT02031198, clinicaltrials.gov) after establishing a favorable safety profile in mild-to-moderate AD patients (phase 1 ID: NCT01850238, clinicaltrials.gov). This is the first active tau immunotherapy candidate tried in humans after showing promising tau clearance profiles in animal models. Antibodies produced by this vaccine showed six-times higher affinity to pathological truncated tau isoform (tau151-391/4R) than to normal full length tau.^135^ The study spanned over 12 weeks, and was randomized in terms of treatment and control (placebo) with a total of 30 (50–58 years old) patients. The patients received a total of 6 monthly subcutaneous injections of AADvac1 or placebo, and patients and caregivers were always blinded to the treatment given. Few patients were withdrawn due to some side effects like injection site reactions and increased micro-hemorrhages. This active immunogen showed remarkable immunogenicity; however, this study had several shortcomings that might affect the interpretation of the safety profiles obtained and the biological cognitive effects. For example, only one dose of the vaccine was used thus making it difficult to determine whether an immune dose-dependent response can be obtained. In addition, and due to the limited number of CSF samples provided by the patients,^136^ no conclusion can be drawn about the magnitude of the effect on tau or AD-related markers’ levels in treated individuals vs. controls. Further studies are needed to determine the clinical effectiveness of ADDvac1. Phase two trial is currently recruiting patients, and the field is hopeful about using this antibody prophylactically.^135, 137^ Other studies utilizing tau immunotherapy are currently in clinical trials, four of which are passive. These studies do not aim at studying the efficacy of the antibody yet, but to only establish a safety profile. One of these studies utilizes a humanized tau-antibody, C2N8E12. It interacts with extracellular form tau aggregate, which makes it’s action independent on cellular entry; thus an major advantage.^79,138^ It was shown to stop tau seeding and trans-neuronal propagation in animal models, and thus potentially stop the progression of AD.^79^ The drug is currently in phase II trials in a study that recruits 400 patients with confirmed AD.^139^ The study will compare three doses of C2N8E12 to placebo, all given over a span of 2 years. In addition to safety, this study will measure the effects of the vaccine on cognitive decline. The study will end in October 2020. (clinicaltrials.gov; NCT02880956).

## Futuristic ideas: combinatorial therapy

A phase III clinical trial with solanezumab, a monoclonal antibody against Aβ16-24, involving 2100 patients with mild AD failed to meet the expected results. However, an A4 (anti-amyloid treatment in asymptomatic AD) clinical study is ongoing with the solanezumab antibody involving older individuals who may be at risk of memory loss. Recently a phase III clinical study of crenezumab, termed CREAD2, evaluating the safety and efficacy of the drug among prodromal to mild AD patients has started (Clinical Trials.gov). As protein aggregation occurs at the early stages of many neurodegenerative diseases, early protein aggregates are most likely the pathogenic forms. Therefore, targeting them at this stage could have beneficial effects as serving as a preventive measure (Fig. 1-a). This could be the explanation of the failure of many immunotherapy trials performed on relatively advanced stage of the disease pathology. With Aβ clinical trials at different stages, this indicate that targeting Aβ alone most likely will not enable the development of a successful therapy.

Abnormal aggregation of α-synuclein, a synaptic protein, has been implicated in a group of neurodegenerative diseases, termed synucleinopathies. α-synuclein pathology has frequently been observed in AD contributing to secondary symptoms. According to the preclinical and clinical studies, immunotherapies against tau, Aβ, and α-synuclein proteins have shown some promising results. Early this year, a global phase II clinical study, PASADNA with PRX002/RG7935 (an anti-alpha-synuclein antibody) involving patients with early Parkinson’s disease has been initiated. A plethora of studies indicate multiple pathogenic proteins interacting in many neurodegenerative disorders. Interaction between tau, Aβ and α-synuclein proteins might affect the pathogenicity of AD, as well. Therefore, a combinatorial immunotherapeutic approach can be designed if the clinical trials of immunotherapy for individual proteins are successful. As each immunotherapy will target different pathogenic proteins at different stages of the disease pathogenesis, such combination therapy might enable us find an effective therapeutic intervention of AD, which can also be extended for many other neurodegenerative diseases (Fig. 1-b).
